# Structural Elucidation and Antioxidant Activities of a Neutral Polysaccharide From Arecanut (*Areca catechu* L.)

**DOI:** 10.3389/fnut.2022.853115

**Published:** 2022-03-07

**Authors:** Xiaolong Ji, Jianhang Guo, Feibing Pan, Fengjun Kuang, Haiming Chen, Xudan Guo, Yanqi Liu

**Affiliations:** ^1^College of Food and Bioengineering, Henan Key Laboratory of Cold Chain Food Quality and Safety Control, Zhengzhou University of Light Industry, Zhengzhou, China; ^2^Huachuang Institute of Areca Research-Hainan, Haikou, China; ^3^Hainan Kouweiwang Science and Technology Development Co., Ltd., Wanning, China; ^4^School of Food Science and Engineering, Hainan University, Haikou, China; ^5^Basic Medical College, Hebei University of Chinese Medicine, Hebei Higher Education Institute Applied Technology Research Center on TCM Formula Preparation, Hebei TCM Formula Preparation Technology Innovation Center, Shijiazhuang, China

**Keywords:** arecanut, purification, polysaccharide, structural characterization, antioxidant activity

## Abstract

A novel neutral polysaccharide designated as PAP1b was isolated from *Areca catechu* L. by hot water extraction, ethanol precipitation, and column chromatography. PAP1b was mainly composed of mannose, galactose, xylose, and arabinose in a ratio of 4.1:3.3:0.9:1.7, with an average molecular weight of 37.3 kDa. Structural characterization indicated that the backbone of PAP1b appeared to be composed mainly of → 6-β-Man*p*-(1 →, → 4)-α-Gal*p*-(1 → and → 3,6)-β-Man*p*-(1 →) residues with some branches, and terminal of (1 →)-linked-β-Man*p* residues. The results of bioactivity experiments showed that PAP1b had antioxidant *in vitro*, esspecially on scavenging DPPH and hydroxyl radicals. Therefore, the polysaccharide from *Areca catechu* L. could be used as a potential antioxidant in functional food.

## Introduction

Arecanut (*Areca chu* L.), which belongs to the family Palmae, is largely cultivated in the subtropical and tropical Asia regions, particularly in China, Philippines, India, Malaysia, Indonesia, *etc*. (1, 2). The arecanut has been cultivated for 1,800 years in China (from Wei and Jin Dynastie), and fruits of *A. catechu* (known as areca nut) were commonly used for chewing products in traditional herbal medicine in *Compendium of Materia Medica* (3). Arecanut contains abundant nutrients: flavonoids, tannins, alkaloids, triterpenes, steroids, fatty acids, polysaccharides, and other active ingredients (1). Modern pharmacology research has demonstrated that arecanut showed different biological activities, including antiparasitic, antifungal, analgesic, anti-allergic effects, and antioxidant, anti-inflammatory, hypoglycemic activity, *etc*. (4–6).

Polysaccharides are natural polymers composed of different monosaccharides and their derivatives connected by different glycosidic bonds (7). Natural polysaccharides have attracted more and more attentions because of their high biological activity and low toxicity (8, 9). Except for the study of Hu et al. (10), no report described the extraction, purification, and structural characterization of areca nut seed polysaccharide (AUP), which was composed of mannose, glucose, galactose, and arabinose with different molar ratios (10). We conjectured that the arecanut polysaccharides might have extraordinary structure and health benefit. Therefore, it is necessary to compare and analyze the morphology, structure, and biological activity of different polysaccharides from *Areca chu* L.

The purpose of this study was to investigate the structural characteristics of a novel neutral polysaccharide from *Areca chu* L. based on high performance gel permeation chromatography (HPGPC), high performance liquid chromatography (HPLC), Fourier transform infrared spectroscopy (FT-IR), methylation analysis, and nuclear magnetic resonance (NMR) spectroscopy. In addition, its antioxidant activity was analyzed by experiments *in vitro*. The results would provide comprehensive information on the structure and biological activity of a neutral polysaccharide from *Areca chu* L. and show its potential as new antioxidant in the functional food and pharmaceutical industry.

## Materials and Methods

### Materials

The arecanut was provided by Hunan Kouweiwang Co., Ltd (Hunan, China). Anion-exchange DEAE Sepharose Fast Flow and Sephacryl S-100 gels were obtained from GE Healthcare Life Sciences (Piscataway, NJ, USA). Standard monosaccharides were originated from Shanghai Aladdin Biochemical Technology Co., Ltd. All other chemicals were analytical grade.

### Extraction, Purification, and Fractionation of Polysaccharides

The extraction of arecanut polysaccharide (AP) was modified according to the reported method (11). Briefly, the recanut was grounded into powder, and the powder was soaked in distilled water (w/v = 1:25) at 90°C for 2 h under ultrasonic treatment. The supernatant was concentrated, and then treated with ethanol (final concentration, 80%) and precipitated at 4°C for 12 h. By centrifugation (3,000 × g, 10 min, 4°C) and freeze-drying to obtain precipitation.

After deproteinization, derosination, decolorization, ethanol precipitation, and freeze-drying, the sample (300 mg) was dissolved in 10-ml distilled water, centrifuged, the supernatant was filtered through a filter, and then injected into a DEAE Sepharose Fast Flow (2.6 × 100 cm) column, which was balanced with distilled water and stepwise gradient NaCl aqueous solution (0.1, 0.2, and 0.3 M) at a flow rate of 0.5 ml/min. The eluent (5 ml/tube) was collected automatically, and the carbohydrate content was determined by the phenol sulfuric acid method. Four components (named AP1, AP2, AP3, and AP4) were obtained, concentrated, and lyophilized for further purification. The 50-mg lyophilized polysaccharide powder was dissolved in 10-ml distilled water and then loaded onto the Sephacryl S-100 gel column (2.6 × 100 cm); the column was eluted with distilled water at a flow rate of 0.6 ml/min and monitored by the phenol sulfuric acid method. As a result, five fine polysaccharide components PAPs (PAP1a, PAP1b, PAP2, PaP3, and PAP4) were obtained. PAP1b was used for further structural characterization and bioassay analysis.

### Physicochemical Property of Polysaccharides

The content of carbohydrates was determined by the phenol sulfuric acid method with glucose as a standard (12). The protein content was estimated by the Bradford method with bovine serum albumin as a reference (11, 13).

### Determination of Homogeneity and Molecular Weight

The uniform distribution and average molecular weight of PAPs were determined by high performance gel permeation chromatography (HPGPC). The HPLC system was equipped with a TSK-GEL G3000PWXL (300 mm × 7.8 mm, i.d.) gel column in series and a Waters 2414 refractive index detector. Elute the sample solution with 0.02 M potassium phosphate solution at a flow rate of 0.6 mL/min at a detector temperature of 35°C. Calibration curves were obtained using standard glucans. The average molecular weight of PAPs was estimated using the calibration curve (14).

### Analysis of Monosaccharide Composition

The monosaccharide compositions of PAPs were determined according to reference (15). In short, PAPs were hydrolyzed with 2 mol/L trifluoroacetic acid in a sealed tube at 120°C for 4 h. The fully hydrolyzed polysaccharide was derived from PMP. On an ODS C18 column (4.6 mm × 250 mm) connected to the Agilent 1260 HPLC system, the derivatives were analyzed at 254 nm. Monosaccharide standards were PMP labeled and analyzed in a similar manner to the above. The monosaccharide composition of PAPs was determined by comparing the retention time of PAP with that of standard. The monosaccharide content was quantified according to the calibration curve of each monosaccharide standard.

### UV-Vis Spectroscopy and FTIR Spectroscopy Analysis

The UV-vis spectra of 1. mg/ml PAP solution was recorded in the range of 200–400 nm using a UV-2450 spectrophotometer (Shimadzu, Japan) (16). The infrared spectra of PAPs were recorded by the KBr disk method and FT-IR spectrometer in the range of 400–4,000 cm^−1^. The absorption peaks were compared and annotated using OMNIC 8.2 software (17).

### Methylation Analysis

In order to determine the glycosyl bond, 30 mg PAP1b was methylated with distilled NaOH/DMSO through the Needs' method, and then methylated with CH_3_I. The reaction was stopped with distilled water. Complete methylation was confirmed by the disappearance of the OH band (3,200–3,700 cm^−1^) in the FT-IR spectrum. Methylated PAP1b was treated with 3 ml of 2-M trifluoroacetic acid at 120°C for 2 h. After removal of formic acid, methylated PAP1b was converted to corresponding aldol acetate by NaBH_4_ reduction at room temperature. The reduced polysaccharide was acetylated with acetic anhydride and then dissolved in chloroform, equipped with an HP-5MS capillary column (30 m × 0.25 mm × 0.25 μm); GC-MS analysis was performed on the GCMS-6890A-5975C instrument (11, 18).

### NMR Spectroscopy

As mentioned earlier, a Bruker 600 MHz Avance spectrometer equipped with an ^1^H/^13^C double probe in the FT mode was used for structural analysis by NMR at 298 K in D_2_O (19). PAP1b was dissolved in deuterium (D_2_O, 99.9%) and frozen three times to replace any exchangeable protons with deuterium. The lyophilized sample was then dissolved in D_2_O, and all spectra were recorded with HOD suppression by presaturation. For the interpretation of ^1^H, ^13^C, ^1^H/^1^H-correlated spectroscopy (COSY), ^1^H/^1^H nuclear overhauser effect spectroscopy (NOESY), ^1^H/^13^C heteronuclear single-quantum coherence (HSQC), and heteronuclear multiple bond coherence (HMBC) spectra were recorded by a state time proportional phase increment for orthogonal detection of indirect dimensions (20).

### Antioxidant Activity *in vitro*

PAP1b was dissolved in deionized water, and the final concentrations were 0.2, 0.4, 0.6, 0.8, 1, 1.5, and 2 mg/ml, respectively. DPPH/hydroxyl radical scavenging capacity, total antioxidant capacity, and reduction capacity were evaluated as described by Ji et al. (11) and Zhang et al. (21).

### Statistical Analysis

All experiments were carried out in triplicate. The results were expressed as mean ± standard deviation (SD), ANOVA, and then Duncan multi-range test. SPSS version 17.0 was used for statistical analysis.

## Results and Discussion

### Extraction, Purification, and Fractionation of Polysaccharides

**Figure 1:**
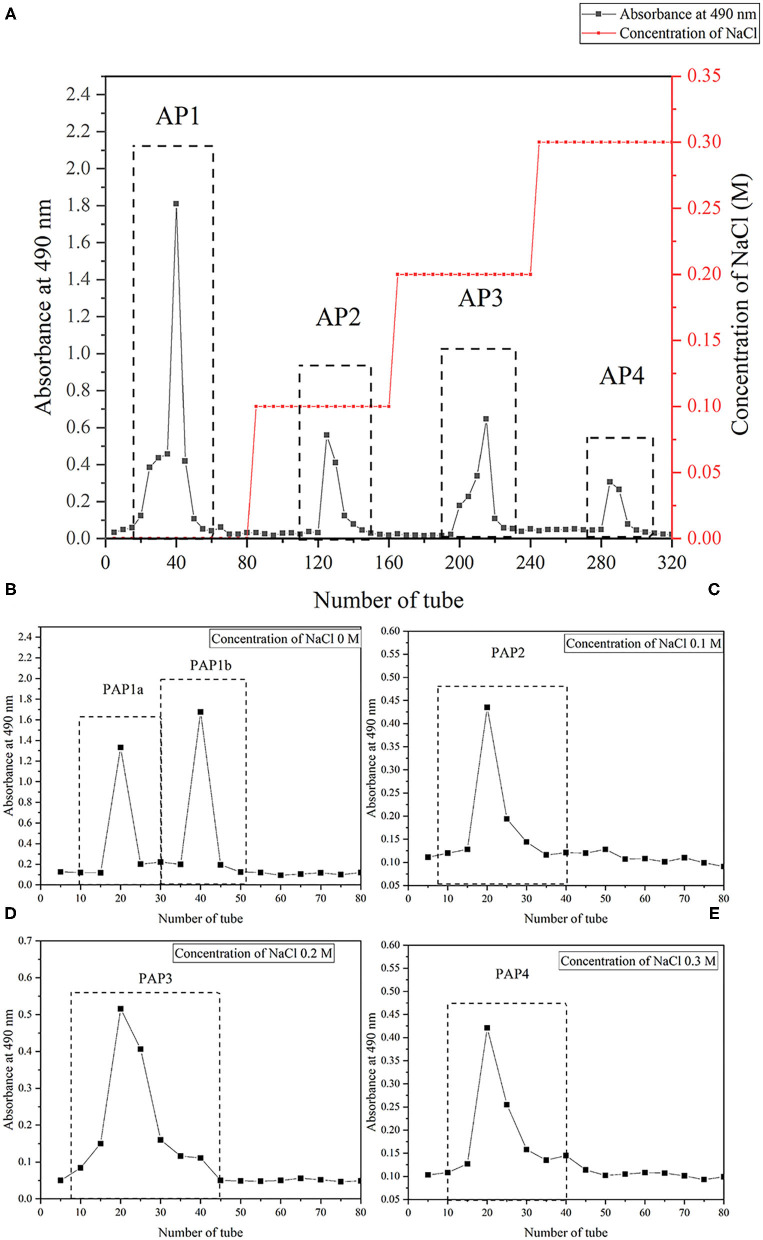
Isolation and purification of crude polysaccharides and five fractions from arecanut (*Areca chu L*.). **(A)** The elution profile of crude arecanut polysaccharides on the DEAE Sepharose Fast Flow (2.6 cm × 100 cm). **(B)** The elution curve of five components PAP1a **(B)**, PAP1b **(B)**, PAP2 **(C)**, PAP3 **(D)**, and PAP4 **(E)** on the Sephacryl S-100 gel column (2.6 cm × 100 cm).

The arecanut (*Areca chu* L.) polysaccharides were obtained by hot water extraction and preliminary purification. Based on dry matter, the yield was 3.73% ± 0.52%. After fractionation by a DEAE Sepharose Fast Flow column, one neutral polysaccharide (AP1) and three acidic polysaccharides (AP2, AP3, and AP4) were produced, and the recoveries were 39.8, 5.2, 6.5, and 5.5%, respectively (Figure 1A). Further purification using a Sephacryl S-100 gel column showed that PAP2, PAP3, and PAP4 displayed a single peak and a symmetrical peak, respectively, and two components (PAP1a and PAP1b) appeared in the elution curve of AP1 (Figure 1B). Therefore, there were five components, PAP1a (yield, 68.21%), PAP1b (83.33%), PAP2 (55.30%) (Figure 1C), PAP3 (37.51%) (Figure 1D), and PAP4 (17.06%) (Figure 1E) for physicochemical properties. The results of chemical analysis showed that the total sugar contents were 8.94, 65.00, 42.5, 19.25, and 40.10%, respectively. UV-vis spectra and the Bradford method (containing 0.58, 0.39, 0.60, 0.63, and 0.64% protein) showed that the five polysaccharide components contained a small amount of protein. In addition, according to the HPGPC method, the average molecular weights (*Mw*) of neutral polysaccharides (PAP1a and PAP1b) were 3.09 × 10^6^ Da and 3.73 × 10^4^ Da, respectively, while the *Mw* of three acidic polysaccharides (PAP2, PAP3, and PAP4) was 5.82 × 10^6^ Da, 3.74 × 10^6^ Da and 3.61 × 10^5^ Da, respectively. The molecular weight of various arecanut (*Areca chu* L.) polysaccharides may be different in the range of 10^4^-10^6^ Da. The *Mw*s of neutral and acidic polysaccharides were similar to coconut inflorescence polysaccharides obtained by Mummaleti et al. (22).

### Monosaccharide Composition

The monosaccharide composition of five arecanut components was analyzed by HPLC. PAP1a was mainly composed of mannose and galactose with a mass ratio of 3:2, and PAP1b composed of mannose, galactose, xylose, and arabinose with a molar ratio of 4.1:3.3:0.9:1.7, indicating that mannose and galactose were the main monosaccharides in neutral polysaccharides. The monosaccharide composition and proportion were similar to those of coconut soluble crude polysaccharides reported by Abbasiliasi et al. (23).

Although the three acidic polysaccharides presented similar monosaccharide profiles, the specific ratios showed significant differences. Mannose, xylose, arabinose, galactose, glucose, and glucuronic acid were the main monosaccharides in PAP2, with the ratio of 1.1:0.8:4.3:2.5:0.7:0.6. The proportion of mannose in PAP3 and PAP4 was higher, and the galactose was lower. Among PAP3 samples, the proportion of arabinose was the highest (34.25%), followed by mannose (26.52%), glucose (14.75%), and galactose (14.32%). However, arabinose (33.93%), mannose (21.43%), galactose (16.07%), and glucose (10.71%) were the main monosaccharides in PAP4. In addition, the contents of uronic acid in PAP2, PAP3, and PAP4 were 6, 6, and 8.93%, respectively. The monosaccharide profiles of the three acid polysaccharides were different from those of pectin and coconut polysaccharides in other studies (24, 25). The above results showed that there were significant differences not only in the monosaccharide spectrum between neutral polysaccharides and acidic polysaccharides but also in the monosaccharide spectrum among the three acidic polysaccharides (26).

### FT-IR Analysis

**Figure 2:**
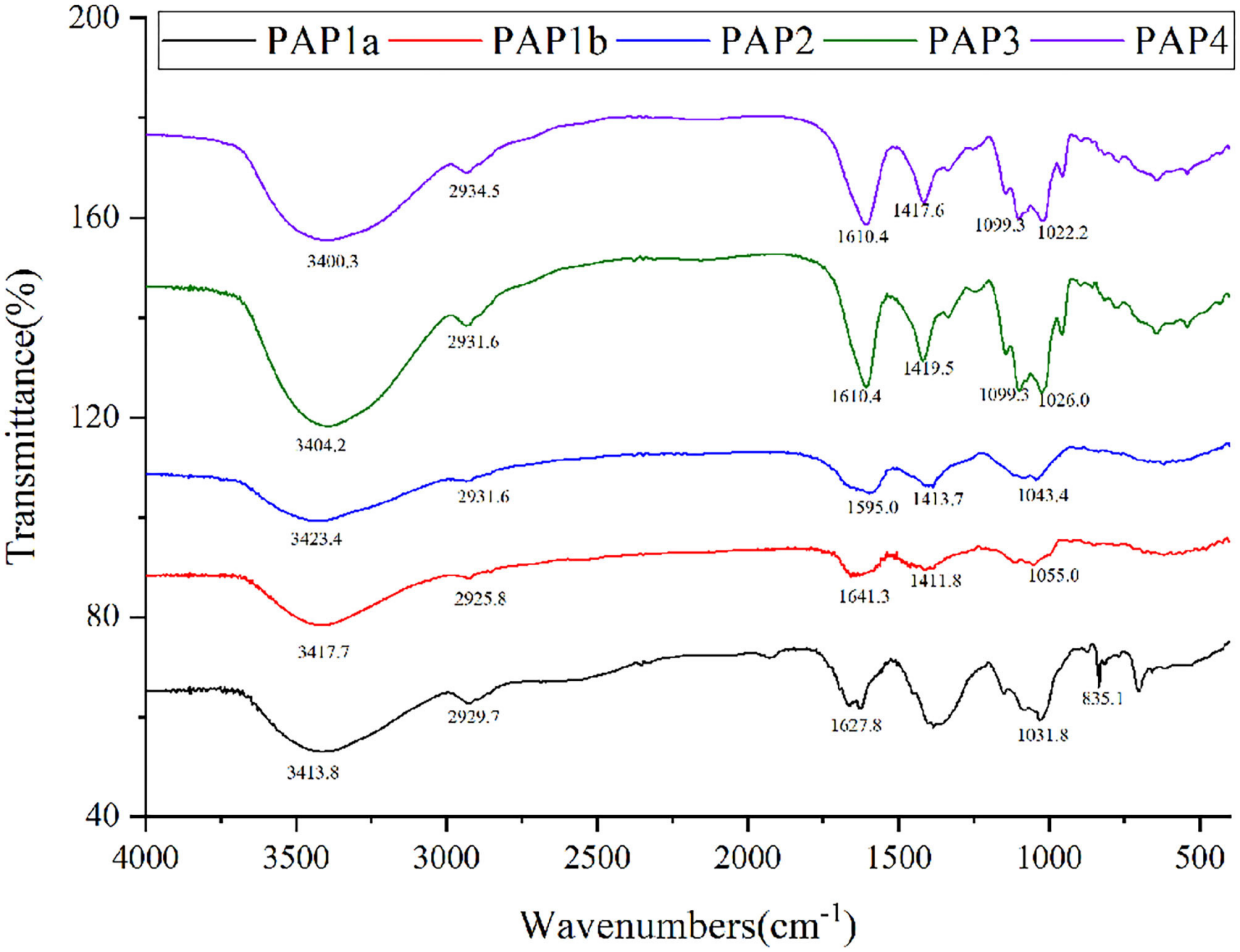
Fourier-transform infrared spectra of five polysaccharides from arecanut (*Areca chu* L.).

The FTIR spectra could provide useful information about the main functional groups of plant polysaccharides (27). Figure 2 showed a wide and strong absorption peak at 3,500–3,400 cm^−1^, which was due to the O-H stretching vibration of intramolecular or intermolecular hydrogen bonds (28, 29). The absorption peak was about 2,930 cm^−1^, which was mainly caused by the asymmetric C-H stretching vibration of CH, CH_2_, and CH_3_ groups. These two absorption peaks were considered to be the characteristic bands of plant polysaccharide polymers (26, 30, 31). Under these two characteristic wavelengths, there were some differences in the absorption intensity of the five polysaccharides. The apparent absorption peaks were located at 1590–1630 cm^−1^ and 1410–1420 cm^−1^; one of the five purified polysaccharides was attributed to the antisymmetric C = O stretching vibration and symmetric C = O stretching vibration of ionic carboxyl (30, 32, 33). The absorption band in the range of 1,000–1,200 cm^−1^ belonged to the C-O-C and O-C-O tensile vibration of glycosidic bonds and rings in polysaccharides or the C-O-H tensile vibration of side groups, in which two/three absorption bands appeared in PAP3 and PAP4, but only one absorption band exists in PAP1a, PAP1b, and PAP2 (7, 34). The absorption peak was located at 835.1 cm^−1^ and appeared in PAP1a, indicating the presence of the areca polysaccharide α-type glycosidic bond (35, 36). The FTIR spectra of these five areca polysaccharides were similar to the corresponding coconut polysaccharides reported by Mummaleti et al., indicating that they had similar structures (22).

### Methylation Analysis of PAP1b

The type and proportion of glycosidic bonds in plant polysaccharide polymers could be understood by methylation analysis (37, 38). Table 1 showed the glycosidic bond pattern in PAP1b, which was the main polysaccharide component. According to the information of total ion chromatography, tandem mass spectrometry and methylated fragments, four methylated sugar derivatives were identified, including t-Man*p*, 6-Man*p*, 4-Gal*p*, and 3,6-Man*p*. The results were similar to those of monosaccharide composition analysis. The structure of PAP1 was further confirmed by NMR.

**Table 1:** Methylation analysis data for PAP1b.

**Peak No**.	**Residues**	**Retention time (min)**	**Methylated sugars**	**Linkage patterns**
1	A	8.940	2,3,4,6-Me_4_-Man*p*	Man*p*-(1 →
2	B	13.746	2,3,4-Me_3_-Man*p*	→ 6)-Man*p*-(1 →
3	C	14.079	2,3,6-Me_2_-Gal*p*	→ 4)-Gal*p*-(1 →
4	D	16.589	2,4-Me_2_-Man*p*	→ 3,6)-Man*p*-(1 →

### NMR Spectroscopy of PAP1b

**Figure 3:**
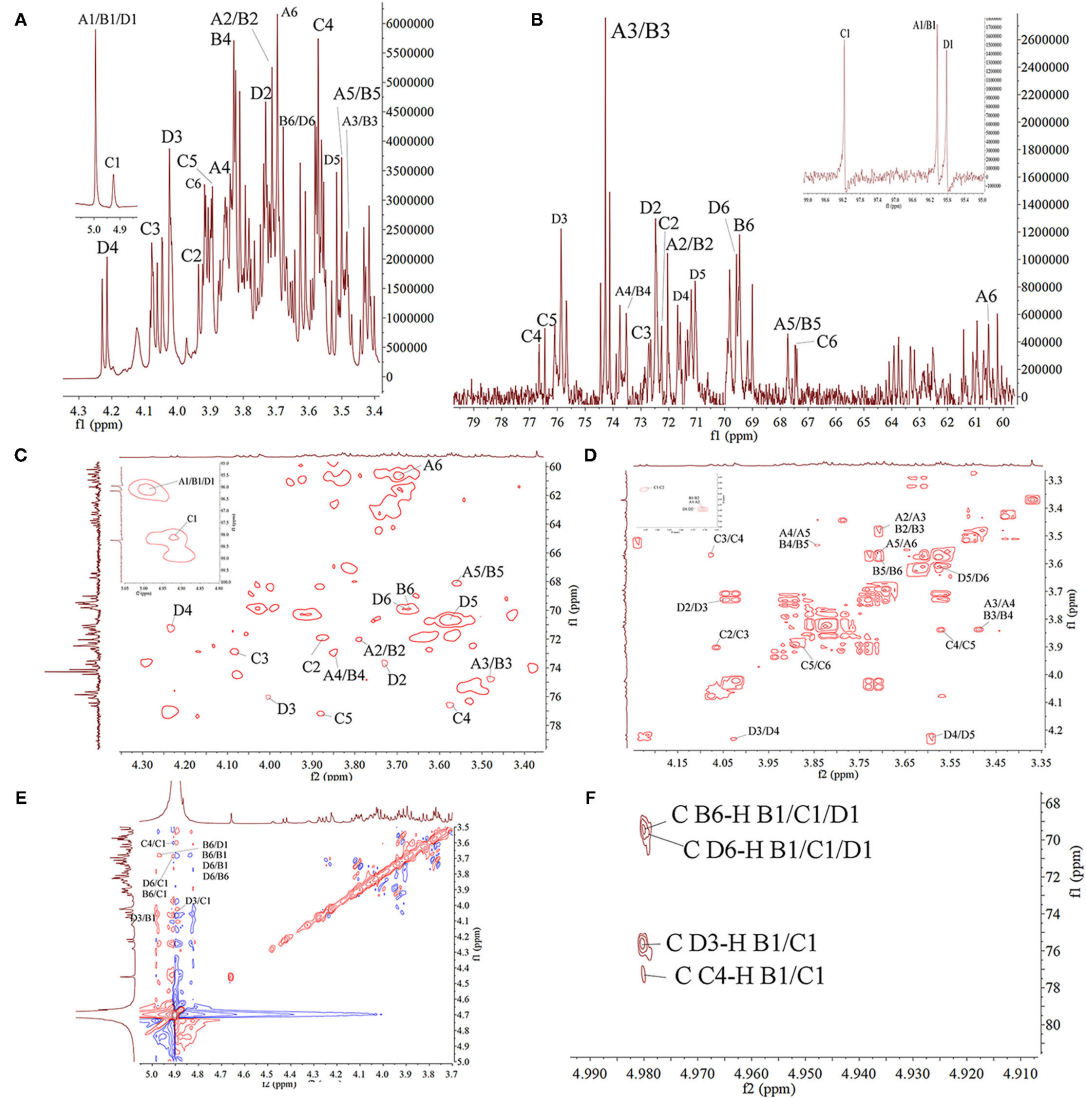
The NMR spectra of PAP1b in D_2_O. **(A)** ^1^H spectra; **(B)** ^13^C spectra; **(C)** HSQC spectra; **(D)** COSY spectra; **(E)** NOESY; **(F)** HMBC spectra.

In order to further clarify the structural characteristics of PAP1b, one-dimensional and two-dimensional NMR spectra were measured and analyzed. In the ^1^H-NMR spectrum (Figure 3A), four main heterogeneous proton signals were observed in PAP1b, which were δ 4.98, 4.98, 4.91, and 4.98, marked A, B, C, and D, respectively. The ^1^H NMR spectra δ chemical shifts in the range of 3.48–4.98 were assigned to protons from residues C-2, C-3, C-4, and C-5. After labeling the corresponding allogeneic carbon signal in the ^13^C-NMR spectrum (Figure 3B), four allogeneic signals δ resonance at 96.04, 96.04, 98.10, and 95.82. According to the results reported in the literature, the signals of all labeled residues in ^1^H and ^13^C NMR spectra were assigned to the data in 2D NMR spectra. Table 2 summarized the proton and carbon assignments of the four main residues in PAP1b based on the chemical shift data obtained from HSQC (Figure 3C), ^1^H-^1^H COSY (Figure 3D), NOESY (Figure 3E), and HMBC (Figure 3F) spectra. The chemical shift of the heteropoly proton of residue A was δH 4.98, and the corresponding signal in the heterogeneous carbon appeared at δC 96.04. The C-2, C-3, C-4, C-5, and C-6 of Residue A produced the signals at δC 72.02/δH 3.71, δC 74.27/δH 3.48, δC 73.52/δH 3.84, δC 67.73/δH 3.55, and δC 60.55/δH 3.70, respectively. According to NMR data, the chemical shift of Residue A was consistent with β-Man*p*-(1 → (39, 40). Similarly, the signals at δC 69.46/δH 3.68 corresponded to the anomeric protons and carbons of Residue B-6, and the residue B was identified as → 6)-β-Man*p*-(1 → (41, 42).

**Table 2:** Assignments of ^1^H and ^13^C NMR spectra for PAP1b.

**Residues**	**Linkage**		**1**	**2**	**3**	**4**	**5**	**6**
A	*β*-Man*p*-(1 →	C	96.04	72.02	74.27	73.52	67.73	60.55
		H	4.98	3.71	3.48	3.84	3.55	3.70
B	→ 6)-*β*-Man*p*-(1 →	C	96.04	72.02	74.27	73.52	67.73	69.46
		H	4.98	3.71	3.48	3.82	3.55	3.68
C	→ 4)-α-Gal*p*-(1 →	C	98.10	72.24	72.65	76.67	76.54	67.46
		H	4.91	3.94	4.08	3.58	3.86	3.91
D	→ 3,6)-*β*-Man*p*-(1 →	C	95.82	72.48	75.86	71.67	71.06	69.57
		H	4.98	3.73	4.02	4.23	3.56	3.68

The cross peak at 69.57–95.82 ppm in the heterospectral region of the HSQC spectrum was, tentatively, Man*p* (Residue D). The chemical shifts of H-1, H-2, H-3, H-4, H-5, and H-6 were obtained from the COSY spectrum at 4.98, 3.73, 4.02, 4.23, 3.56, and 3.68 ppm, respectively, which was consistent with other reports (43). According to the chemical shift of protons in the HSQC spectrum, the carbon chemical shift of Residue C from C-1 to C-6 was determined (Table 2). The up-field shift of C-2 (72.48 ppm) and down-field C-4 (71.67 ppm) showed that Residue D was 1,3,5-trisubstituted mannose. The signals at 72.48 and 71.67 ppm were assigned to C-2 and C-4 of Residue D, respectively. For 3-*O*-acetyl derivatives, the up-shift field of C-2 and the down-shift field of C-4 were significantly higher than that of C-2 non-acetylated Man*p*. Therefore, it could be inferred that the acetyl group was connected to the C-3 position of Man*p* residue. Then, the cross peaks of C/H-2 protons (72.48/3.73) and C/H-4 protons (71.67/4.23) of Residue D further confirmed this result. These results were consistent with the results of methylation and FT-IR analysis, indicating that Residue D was 3-*O*-acetyl-(1 → 6)-linked Man*p* (44, 45). The chemical shift of the heteropoly proton of residue C was δ 4.91, and the corresponding chemical shift in heteropoly carbon was δ 98.10. The other protons of Residue A were assigned according to the COSY spectrum. Other corresponding carbon and hydrogen signals were determined by HSQC with δ 72.24 (3.94), 72.65 (4.08), 76.67 (3.58), 76.54 (3.86), and 67.46 (3.91). Based on these NMR data, we infer that 1,4-Gal*p* (46, 47).

The COSY and HMBC could determine the glycosidic bond between sugar residues (48). Therefore, using these techniques, the residue internal connection was determined and listed in Table 2. As shown in the HMBC spectrum, some residual cross peaks were identified: C D-6 to H B-1/C-1/D1, C B-6 to H B-1/C-1/D1, C D-3 to H B-1/C-1, and C C-4 to H B-1/C-1. In addition, some residual cross peaks were identified in the COSY spectrum: A/B/C/D H-1 to A/B/C/D H-2, A/B/C/D H-2 to A/B/C/D H-3, A/B/C/D H-3 to A/B/C/D H-4, A/B/C/D H-4 to A/B/C/D H-5, and A/B/C/D H-5 to A/B/C/D H-6; and in the NOESY spectrum: B H-6 to D/B H-1, D H-6 to D/B H-1, C H-4/D H-6/B H-6/D H-3 to C H-1, and D H-3 to B H-1.

According to the monosaccharide composition of PAP1b, with the analysis results of FT-IR, GC-MS and 1D/2D NMR, it was determined that PAP1b was mainly composed of the → 4)-α-Gal*p*-(1 → and → 6)-β-Man*p*-(1 → backbone with a branch point at *O*-3.

### Antioxidant Activity *in vitro* of PAP1b

Imbalance of reactive oxygen species could lead to many metabolic disorders and diseases, such as cancer, hypertension, and diabetes (49). It has been reported that plant polysaccharides could protect the body from oxidative damage (50, 51). We evaluated the antioxidant activity of PAP1b *in vitro* by DPPH/hydroxyl radical scavenging, Fe^2+^ chelating activity, and total reduction ability (21, 52, 53).

**Figure 4:**
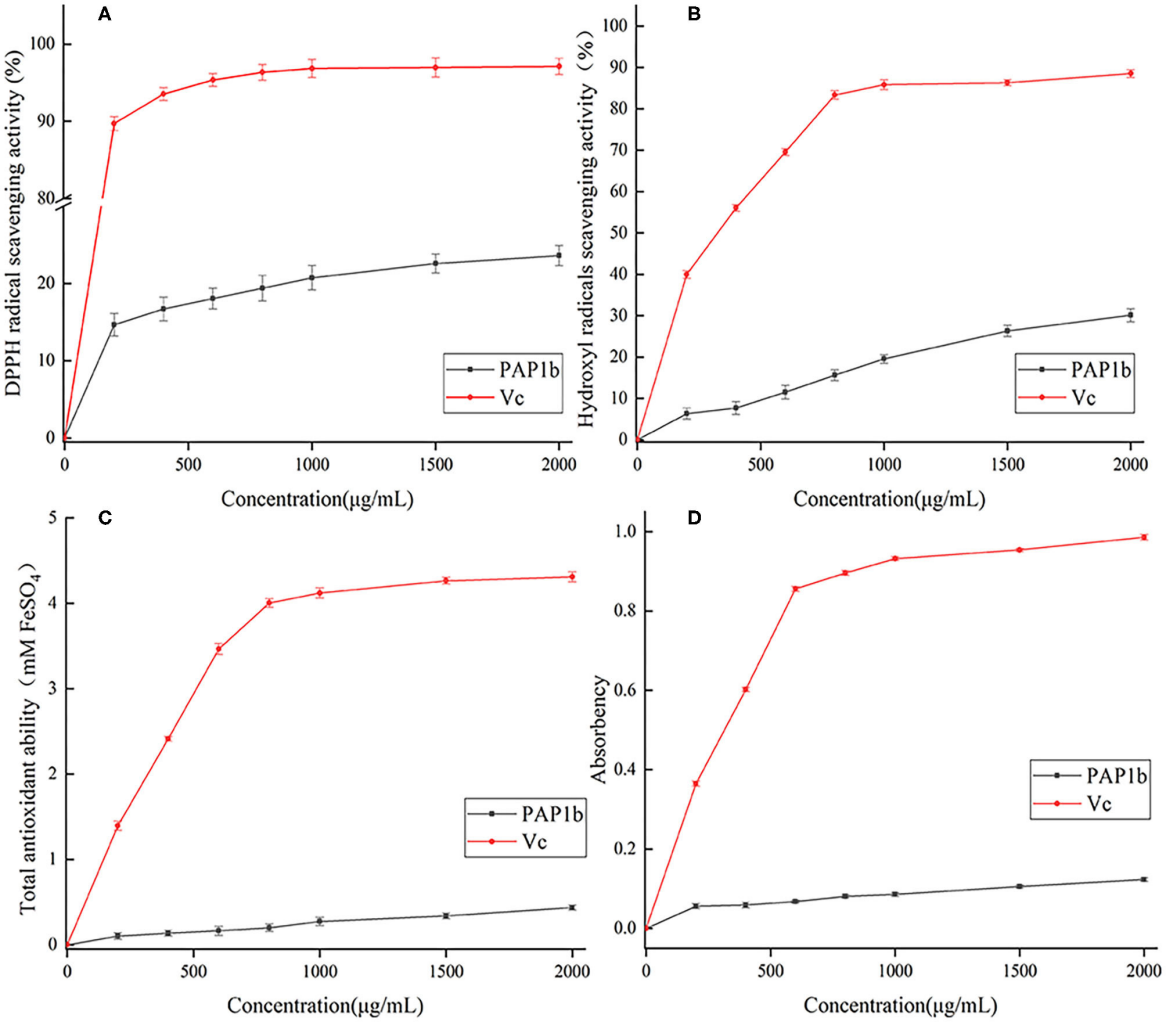
Antioxidant activity of PAP1b: **(A)** the scavenging activity of DPPH radical; **(B)** the scavenging activity of hydroxyl radical; **(C)** total antioxidant ability; **(D)** the chelating activity of Fe^2+^.

DPPH • free radical was considered as a simple model to detect the antioxidant activity of polysaccharides *in vitro* (54). As shown in Figure 4A, in the range of 0–2 mg/ml, the ability of PAP1b to scavenge DDPH free radicals gradually increased and was lower than that of vitamin C. In addition, at the concentration of 2 mg/ml, the DPPH free radical scavenging activity was 23.61%, which was lower than the polysaccharide content obtained from *Medemia argun* fruit by water extraction (55). The generation of superoxide anion binding to hydroxyl (-OH) may lead to DNA damage and destroy human function (56). Figure 4B showed the scavenging ability of PAP1b to hydroxyl radicals, and PAP1b exhibited a dose-response relationship. The clearance rate of PAP1b at the concentration of 2 mg/ml was 30.14%, which was almost the same as that of Vc at the concentration of 0.2 mg/ml (39.97%). The antioxidant activity of PAP1b to hydroxyl radical might be due to the improvement of hydrogen supply capacity of PAP1b to hydroxyl radical (57).

The FRAP method was used to measure the total antioxidant capacity of plant polysaccharide samples and based on the measurement of TPTZ-Fe(II) complex produced by reducing TPTZ-Fe(III) complex by plant polysaccharide. The corresponding FeSO_4_ values were calculated using standard curves and regression equations (53). The higher the FeSO_4_ value, the higher the iron reduction capacity (58). As shown in Figure 4C, the total antioxidant capacity of PAP1b and Vc depended on the concentration. The activity of PAP1b was significantly lower than that of V_C_ (*p*< 0.05). At 2 mg/ml, the total antioxidant activity of PAP1b was determined to be equivalent to 0.43 mm FeSO_4_, and V_C_ was 4.31-mm FeSO_4_. The ability of polysaccharide to reduce Fe^3+^ to Fe^2+^ was investigated by reducing the force method. The absorbance at 700 nm was measured to describe the reduction ability of natural compounds; the greater the value of A700, the better the reduction capacity (59, 60). As shown in Figure 4D, the reducing power of PAP1b increased slowly in concentration, V_C_ increased rapidly, and then remains unchanged; the maximum absorbance value of V_C_ was.952. When the concentration of PAP1b reached 2 mg/ml, the absorbance (0.123) was lower than the standard V_C_.

The antioxidant activity and non-toxic characteristics of arecanut polysaccharides (PAP1b) could make it possess potential application value in food and pharmaceutical industry. The exact mechanism *in vivo* and relationships between polysaccharide structure and antioxidant activity is one of our future research topics.

## Conclusion

Five polysaccharides were successfully isolated and purified from arecanut (*Areca chu* L.), including two neutral polysaccharides (PAP1a and PAP1b) and three acidic polysaccharides (PAP2, PAP3, and PAP4). They differed in monosaccharide composition (mainly type rather than the mass ratio) and average molecular weight. PAP1b was mainly composed of mannose, galactose, xylose, and arabinose, with a ratio of 4.1:3.3:0.9:1.7 and an average molecular weight of 3.73 × 10^4^ Da. PAP1a, PAP2, PAP3, and PAP4 were homogeneous heteropolysaccharides with different monosaccharide compositions and molecular weights. The backbone of PAP1b appeared to be mainly composed of → 6)-β-Man*p*-(1 →, → 4)-α-Gal*p*-(1 → and → 3,6)-β-Man*p*-(1 → residues with some branches and terminals of (1 →)-linked-β-Man*p* residue. Furthermore, the antioxidant activity of PAP1b *in vitro* was dose dependent. These results could provide a theoretical basis for further research on the antioxidant activity *in vivo* and structure-activity relationship.

## Data Availability Statement

The original contributions presented in the study are included in the article/supplementary material, further inquiries can be directed to the corresponding author/s.

## Author Contributions

XJ contributed to conception, design, and funding of the study. JG, FP, and FK organized the database. HC wrote the first draft of the manuscript. XG and YL contributed to writing, review, and editing. All the authors contributed to the article and approved the submitted version.

## Funding

This work was funded by Dean of Huachuang Institute of Areca Research-Hainan (HCBL2020YZ-010), Basic Research Plan of Higher Education School Key Scientific Research Project of Henan Province (21A550014), and Doctoral Research Foundation of Zhengzhou University of Light Industry (2020BSJJ015), and Science and Technology Research Project of Higher Education in Hebei Province (QN2020233).

## Conflict of Interest

FK was employed by Hainan Kouweiwang Science and Technology Development Co., Ltd. The remaining authors declare that the research was conducted in the absence of any commercial or financial relationships that could be construed as a potential conflict of interest.

## Publisher's Note

All claims expressed in this article are solely those of the authors and do not necessarily represent those of their affiliated organizations, or those of the publisher, the editors and the reviewers. Any product that may be evaluated in this article, or claim that may be made by its manufacturer, is not guaranteed or endorsed by the publisher.
